# Supplementary material for: Prevalence of keratoconus and keratoconus suspect, and their characteristics on corneal tomography in a population-based study
Source: PLoS One. 2025 Jan 16;20(1):e0308892. doi: 10.1371/journal.pone.0308892 (PMC11737720; doi:10.1371/journal.pone.0308892)
Supplement: S1 File — (DOCX) [file pone.0308892.s001.docx]

STROBE Statement—checklist of items that should be included in reports of observational studies

|  | Item No. | Recommendation | Page  No. | Relevant text from manuscript |
| --- | --- | --- | --- | --- |
| **Title and abstract** | 1 | (*a*) Indicate the study’s design with a commonly used term in the title or the abstract | *2* | *We aimed to determine the prevalence and corneal tomographic characteristics of KC and keratoconus suspect (KCS) in a population-based study,* |
|  |  | (*b*) Provide in the abstract an informative and balanced summary of what was done and what was found | *2* | *Systemic and ophthalmological examinations including corneal tomography with swept-source anterior segment optical coherence tomography (AS-OCT) were conducted to determine the prevalence and corneal tomographic characteristics of KC and KCS. In addition, data on 766 eyes were used to construct discrimination models with or without corneal tomography. In results, KC was diagnosed in six (0.85%) participants, and KCS was diagnosed in 27 (1.46%) participants. The values including corneal power, keratometric cylinder, corneal central and thinnest thickness, corneal asymmetry, higher-order irregularity, and their inter-eye differences were associated with KC and KCS. The areas under the receiver operating characteristic curves for the three multivariate discrimination models (without corneal tomography, with corneal tomography, and without corneal tomography + inter-eye difference models) for participants with KC or KCS were 0.848, 1.000, and 0.930, respectively.* |
| Introduction | | | |  |
| Background/rationale | 2 | Explain the scientific background and rationale for the investigation being reported | *4-5* | *KC is typically diagnosed after corneal irregular astigmatism has become clinically significant. However, the development of the Placido-based corneal topographer [25], scanning [26], the Scheimpflug-based corneal tomographer [17,27,28], and anterior segment optical coherence tomography (AS-OCT) [29–31] have enabled clinicians to detect not only clinical KC, but also keratoconus suspect (KCS), and forme fruste KC [26,32–35]. However, corneal tomography using Scheimpflug camera or AS-OCT is not always available in the clinic.*  *The reported prevalence of KC varies with a range of environmental, genetic, ethnic factors and the instruments, diagnostic criteria, and study designs [8–20]. For example, Kennedy et al. reported a disease prevalence of approximately 54.5 per 100,000 people in the United States based on conventional examinations [9], which differed from the reported prevalence in Israel (2.34%) [14] and Iran (0.76%) [17] where modern corneal topography/tomography was used.* |
| Objectives | 3 | State specific objectives, including any prespecified hypotheses | *5* | *Therefore, this study determined the prevalence of KC using corneal tomography, investigated the corneal tomographic characteristics of KC and KCS, and constructed predictive models of KC and KCS with or without corneal tomography.* |
| Methods | | | |  |
| Study design | 4 | Present key elements of study design early in the paper | *5* | *This study was conducted as part of the Yamagata Study, a population-based epidemiological investigation examining systemic and ophthalmologic disorders in Japanese individuals aged ≥ 35 years. Details regarding the study participants and research methods used in this study have been described previously [40–44].* |
| Setting | 5 | Describe the setting, locations, and relevant dates, including periods of recruitment, exposure, follow-up, and data collection | *5, 6* | *Briefly, systemic and ophthalmic data were obtained from residents living in Funagata Town between 2015 and 2017.*  *Refractive spherical and cylindrical power, corneal cylindrical power, and intraocular pressure (IOP) were measured using an auto-ref/kerato/tonometer (TONOREF II, Nidek Co., Ltd., Aichi, Japan). Axial length was measured using a swept-source optical coherence tomography (OCT)-based biometer (OA-2000, TOMEY Corp., Aichi, Japan). Corneal tomography was performed through swept-source AS-OCT (CASIA SS-1000: 2015 examinations and CASIA2 SS-2000: 2016 and 2017 examinations, Tomey Corporation; Aichi, Japan) [44–46]. Physical characteristics such as height and weight were also recorded.* |
| Participants | 6 | (*a*) *Cohort study*—Give the eligibility criteria, and the sources and methods of selection of participants. Describe methods of follow-up  *Case-control study*—Give the eligibility criteria, and the sources and methods of case ascertainment and control selection. Give the rationale for the choice of cases and controls  *Cross-sectional study*—Give the eligibility criteria, and the sources and methods of selection of participants | *5-6* | *Person- and eye-specific investigations were performed on the participants. The participant selection process is presented in Figs 1 and 2.*  *Fig 1. Flowchart of the data-filtering procedure in person-specific investigations. A total of 822 participants were included in this study.*  *Fig 2. Flowchart of the data-filtering procedure in eye-specific investigations. A total of 1,544 eyes were included in the eye-specific analyses.* |
|  |  | (*b*) *Cohort study*—For matched studies, give matching criteria and number of exposed and unexposed  *Case-control study*—For matched studies, give matching criteria and the number of controls per case |  |  |
| Variables | 7 | Clearly define all outcomes, exposures, predictors, potential confounders, and effect modifiers. Give diagnostic criteria, if applicable | *6-8* | *KC and KCS were diagnosed by two experienced corneal specialists (HN and NM). KC was defined as eyes that showed visual acuity less than 20/20 and keratoconus pattern in axial power maps of the anterior corneal topography such as abnormal localized steepening and / or an asymmetric skewed bow-tie pattern. Additionally, the presence of abnormal elevation on the posterior surface and corneal thinning at the cone was confirmed (Fig 3). KCS was defined as eyes that had visual acuity 20/20 or better and keratoconus patterns in anterior axial power map (Fig 4) [3,31-34].*  *Tomographic patterns were evaluated to analyze the corneal shapes among eyes with KC/KCS and control eyes, as described by Fuchihata et al. [49]. The axial power maps were classified into eight patterns: round, oval, symmetric bowtie, asymmetric bowtie, central steepening, lazy eight, inferior steepening, and crab claw. The definition of “central steepening” was K-readings > 47.0 diopters (D), which is outside the mean plus two standard deviations, in addition to the topographic pattern. Elevation maps of the anterior and posterior surfaces were categorized into nine patterns: central regular ridge, central irregular ridge, central incomplete ridge, central island, asymmetric regular ridge, asymmetric irregular ridge, asymmetric incomplete ridge, asymmetric island, and unclassified. Pachymetric maps were classified into six patterns: central round, central oval, paracentral round, paracentral oval, decentered round, and decentered oval. The term “central” was used when the thinnest point was located within the central 2-mm-diameter zone of the cornea. The map was classified as paracentral when the point was beyond the 2-mm-diameter zone and within a 3-mm-diameter zone. Similarly, the map was classified as decentered if the point was outside the 3-mm diameter zone.*  *Fourier analysis was performed using CASIA/CASIA2 software to evaluate corneal irregular astigmatism. Corneal dioptric data were expanded into spherical power, asymmetry components (first-order harmonic), regular astigmatism (second-order harmonic), and higher-order irregularity (HOI) components (third-and higher-order harmonics) within the central 6-mm zone were analyzed [47,48].* |
| Data sources/ measurement | 8* | For each variable of interest, give sources of data and details of methods of assessment (measurement). Describe comparability of assessment methods if there is more than one group | *7-8* | *The axial power maps were classified into eight patterns: round, oval, symmetric bowtie, asymmetric bowtie, central steepening, lazy eight, inferior steepening, and crab claw. The definition of “central steepening” was K-readings > 47.0 diopters (D), which is outside the mean plus two standard deviations, in addition to the topographic pattern. Elevation maps of the anterior and posterior surfaces were categorized into nine patterns: central regular ridge, central irregular ridge, central incomplete ridge, central island, asymmetric regular ridge, asymmetric irregular ridge, asymmetric incomplete ridge, asymmetric island, and unclassified. Pachymetric maps were classified into six patterns: central round, central oval, paracentral round, paracentral oval, decentered round, and decentered oval. The term “central” was used when the thinnest point was located within the central 2-mm-diameter zone of the cornea. The map was classified as paracentral when the point was beyond the 2-mm-diameter zone and within a 3-mm-diameter zone. Similarly, the map was classified as decentered if the point was outside the 3-mm diameter zone.*  *Fourier analysis was performed using CASIA/CASIA2 software to evaluate corneal irregular astigmatism. Corneal dioptric data were expanded into spherical power, asymmetry components (first-order harmonic), regular astigmatism (second-order harmonic), and higher-order irregularity (HOI) components (third-and higher-order harmonics) within the central 6-mm zone were analyzed [47,48].*  *The inter-eye differences (IEDs) in corneal parameters were determined by subtracting each variable's lower value from the higher value.* |
| Bias | 9 | Describe any efforts to address potential sources of bias | *5-6* | *The participant selection process is presented in Figs 1 and 2.*  *Fig 1. Flowchart of the data-filtering procedure in person-specific investigations. A total of 822 participants were included in this study.*  *Fig 2. Flowchart of the data-filtering procedure in eye-specific investigations. A total of 1,544 eyes were included in the eye-specific analyses.* |
| Study size | 10 | Explain how the study size was arrived at | *5* | *Details regarding the study participants and research methods used in this study have been described previously [40–44]. Briefly, systemic and ophthalmic data were obtained from residents living in Funagata Town between 2015 and 2017.* |

Continued on next page

| Quantitative variables | 11 | Explain how quantitative variables were handled in the analyses. If applicable, describe which groupings were chosen and why | *6, 8* | *Refractive spherical and cylindrical power, corneal cylindrical power, and intraocular pressure (IOP) were measured using an auto-ref/kerato/tonometer (TONOREF II, Nidek Co., Ltd., Aichi, Japan). Axial length was measured using a swept-source optical coherence tomography (OCT)-based biometer (OA-2000, TOMEY Corp., Aichi, Japan). Corneal tomography was performed through swept-source AS-OCT (CASIA SS-1000: 2015 examinations and CASIA2 SS-2000: 2016 and 2017 examinations, Tomey Corporation; Aichi, Japan) [44–46]. Physical characteristics such as height and weight were also recorded.*  *Fourier analysis was performed using CASIA/CASIA2 software to evaluate corneal irregular astigmatism. Corneal dioptric data were expanded into spherical power, asymmetry components (first-order harmonic), regular astigmatism (second-order harmonic), and higher-order irregularity (HOI) components (third-and higher-order harmonics) within the central 6-mm zone were analyzed [47,48].* |
| --- | --- | --- | --- | --- |
| Statistical methods | 12 | (*a*) Describe all statistical methods, including those used to control for confounding | *8-9* | *All statistical analyses were performed using STATA (version 14.2; StataCorp LLC, College Station, TX, USA) and SPSS (version 21.0; IBM Corp., Armonk, NY, USA) statistical software. Statistical significance was set at p <0.05. To compare person-specific and eye-specific characteristics, statistical tests were performed between non-KC, KCS, and KC groups. To determine the risk factors and discrimination models, non-KC and KC/KCS groups were included in statistical investigations. Clopper–Pearson exact confidence intervals (CIs) were evaluated to calculate 95% CIs for the prevalence of KC and KCS. A one-way analysis of variance and Tukey–Kramer tests were performed to compare person-specific characteristics within the non-KC, KCS, and KC groups. Kruskal–Wallis and Dunn–Bonferroni tests were used to estimate eye-specific characteristics. In corneal tomographic and pachymetric patterns investigations, the Chi-square test was used to compare distributions in the non-KC, KCS, and KC groups. Univariate logistic regression analyses were performed to determine the risk factors for KC and KCS. The odds ratios (ORs) and 95% CIs were calculated for each factor. The inter-eye differences (IEDs) in corneal parameters were determined by subtracting each variable's lower value from the higher value. The IEDs of the non-KC and KC/KCS groups were compared using the Mann–Whitney test. Multivariate discrimination models were established based on the identified risk factors for diagnosing KC/KCS. Receiver operating characteristic (ROC) curves of the discrimination models were drawn, and the area under the ROC curve (AUC) values were calculated to assess the sensitivity and specificity of the models. The cutoff point on the ROC curve was determined using the Youden index.* |
|  |  | (*b*) Describe any methods used to examine subgroups and interactions | *8* | *To compare person-specific and eye-specific characteristics, statistical tests were performed between non-KC, KCS, and KC groups. To determine the risk factors and discrimination models, non-KC and KC/KCS groups were included in statistical investigations.* |
|  |  | (*c*) Explain how missing data were addressed | *5-6* | *The participant selection process is presented in Figs 1 and 2.*  *Fig 1. Flowchart of the data-filtering procedure in person-specific investigations. A total of 822 participants were included in this study.*  *Fig 2. Flowchart of the data-filtering procedure in eye-specific investigations. A total of 1,544 eyes were included in the eye-specific analyses.* |
|  |  | (*d*) *Cohort study*—If applicable, explain how loss to follow-up was addressed  *Case-control study*—If applicable, explain how matching of cases and controls was addressed  *Cross-sectional study*—If applicable, describe analytical methods taking account of sampling strategy | *5* | *Details regarding the study participants and research methods used in this study have been described previously [40–44]. Briefly, systemic and ophthalmic data were obtained from residents living in Funagata Town between 2015 and 2017.* |
|  |  | (*e*) Describe any sensitivity analyses | *8-9* | *Multivariate discrimination models were established based on the identified risk factors for diagnosing KC/KCS. Receiver operating characteristic (ROC) curves of the discrimination models were drawn, and the area under the ROC curve (AUC) values were calculated to assess the sensitivity and specificity of the models. The cutoff point on the ROC curve was determined using the Youden index.* |
| Results | | | | |
| Participants | 13* | (a) Report numbers of individuals at each stage of study—eg numbers potentially eligible, examined for eligibility, confirmed eligible, included in the study, completing follow-up, and analysed | *5-6, 9, 10, 16* | *The participant selection process is presented in Figs 1 and 2.*  *Fig 1. Flowchart of the data-filtering procedure in person-specific investigations. A total of 822 participants were included in this study.*  *Fig 2. Flowchart of the data-filtering procedure in eye-specific investigations. A total of 1,544 eyes were included in the eye-specific analyses.*  *A total of 822 participants (378 males and 444 females) were included in this study (Table 1).*  *A total of 1,544 eyes were included in the eye-specific analysis of corneal tomographic patterns, and 392 eyes were excluded due to pterygia or insufficient data.*  *A total of 766 eyes were included in the eye-specific analysis of the risk associations with KC/KCS.* |
|  |  | (b) Give reasons for non-participation at each stage | *5-6* | *The participant selection process is presented in Figs 1 and 2. Because of their analogue, figure-based estimations, the tomographic patterns were investigated in eyes examined both by SS-1000 and by SS-2000. Nevertheless, the risk associations with KC/KCS were investigated only in eyes examined by SS-2000 to avoid numerical differences from the devices.*  *Fig 1. Flowchart of the data-filtering procedure in person-specific investigations. A total of 822 participants were included in this study.*  *Fig 2. Flowchart of the data-filtering procedure in eye-specific investigations. A total of 1,544 eyes were included in the eye-specific analyses.* |
|  |  | (c) Consider use of a flow diagram | *6* | *Fig 1. Flowchart of the data-filtering procedure in person-specific investigations. A total of 822 participants were included in this study.*  *Fig 2. Flowchart of the data-filtering procedure in eye-specific investigations. A total of 1,544 eyes were included in the eye-specific analyses.* |
| Descriptive data | 14* | (a) Give characteristics of study participants (eg demographic, clinical, social) and information on exposures and potential confounders | *9* | *A total of 822 participants (378 males and 444 females) were included in this study (Table 1). KC was detected in at least one eye in seven participants (0.85%, 95% CI: 0.41%–1.78%), and KCS was detected in at least one eye in 12 participants (1.46%, 95% CI: 0.82%–2.56%). The mean participant age was 62.3 ± 11.2 years, height was 159.1 ± 9.4 cm, and weight was 60.6 ± 11.5 kg (Table 1).* |
|  |  | (b) Indicate number of participants with missing data for each variable of interest | *6* | *Fig 1. Flowchart of the data-filtering procedure in person-specific investigations. A total of 822 participants were included in this study.*  *Fig 2. Flowchart of the data-filtering procedure in eye-specific investigations. A total of 1,544 eyes were included in the eye-specific analyses.* |
|  |  | (c) *Cohort study*—Summarise follow-up time (eg, average and total amount) |  |  |
| Outcome data | 15* | *Cohort study*—Report numbers of outcome events or summary measures over time |  |  |
|  |  | *Case-control study—*Report numbers in each exposure category, or summary measures of exposure |  |  |
|  |  | *Cross-sectional study—*Report numbers of outcome events or summary measures | *9, 10, 16, 20* | *Demographic characteristics and prevalence of KC and KCS*  *Corneal tomographic and pachymetric patterns*  *Multivariate discrimination model for diagnosing KC/KCS* |
| Main results | 16 | (*a*) Give unadjusted estimates and, if applicable, confounder-adjusted estimates and their precision (eg, 95% confidence interval). Make clear which confounders were adjusted for and why they were included | *9* | *Although the mean participant age had significant difference in the analysis of variance, it was not considered as a confounder in subsequent investigations. Because KC is a young-onset disease, KC may have already developed until 35 years.* |
|  |  | (*b*) Report category boundaries when continuous variables were categorized |  |  |
|  |  | (*c*) If relevant, consider translating estimates of relative risk into absolute risk for a meaningful time period |  |  |

Continued on next page

| Other analyses | 17 | Report other analyses done—eg analyses of subgroups and interactions, and sensitivity analyses | *21* | *The details of these models are described in Table 8 and Fig 5. The sensitivity and specificity of the non-OCT model were 92% and 79%, respectively. The AUC of the non-OCT model was 0.85. The OCT model had a sensitivity of 100% and a specificity of 100%. The AUC of the OCT model was 1.0. The sensitivity of the non-OCT + IED model was 89%, specificity was 90%, and AUC was 0.90. All models could discriminate eyes with KC effectively (n=5 in eyes examined using SS-2000 in 2016 or 2017).* |
| --- | --- | --- | --- | --- |
| Discussion | | | | |
| Key results | 18 | Summarise key results with reference to study objectives | *22-23* | *This study provided the prevalence of KC (0.85%) and KCS (1.46%) and confirmed the tomographic and pachymetric patterns of the Japanese participants in each group. In addition, we investigated the risk factors for KC/KCS and constructed a discrimination model for predicting these diseases with and without corneal tomography.* |
| Limitations | 19 | Discuss limitations of the study, taking into account sources of potential bias or imprecision. Discuss both direction and magnitude of any potential bias | *27-28* | *This study also has several limitations. First, the population size may have been small to investigate the prevalence of KC effectively. In addition, the CIs were imprecise; for example, the CI for KC prevalence ranged from 0.41 to 1.78%. Additional research conducted using a larger population is necessary. Second, the prevalence of KCS (1.46%), may have been underestimated due to the diagnostic criteria. Our data included a number of aged participants who might have had cataract or other diseases influencing visual acuity. Actually, some patients who had mild keratoconus patterns in axial power maps and visual acuities less than 20/20 were excluded from our analyses because of an “abnormal corneal shape.” Third, participants aged < 35 years were not included in this study. This is an inherent limitation of the study design that only used examination data obtained from medical checkups for detecting adult diseases. However, the incidences of KC/KCS in young population may be underestimated, as cases that will progress to KC or KCS are not included. Therefore, the estimation of prevalence of KC/KCS in 35 years or older may be more effective for investigating the prevalence of KC or KCS. The high prevalence of KC indicated by this study’s findings will help better understand and develop treatment strategies for KC/KCS in Japan. On the other hands, studies conducted in different populations or ethnicities may yield different results, and similar studies are required for various settings, populations, and ethnicities.* |
| Interpretation | 20 | Give a cautious overall interpretation of results considering objectives, limitations, multiplicity of analyses, results from similar studies, and other relevant evidence | *23-26* | *The reported prevalence of KC varies greatly [8–20], one of which may be due to geographic differences. Pearson et al. reported that the prevalence of KC in Asian populations (229 per 100,000) is approximately four times that in Caucasian populations (57 per 100,000) [8]. The prevalence of KC in Middle Eastern, Egyptian, and Indian populations has been reported to be approximately 10- to 50-fold higher than that in European populations [14–17]. Elbedewy et al. speculated that the high prevalence of KC in the Middle East might be associated with the high rate of chronic allergies and vernal keratoconjunctivitis in its populations [16]. In addition, continuous eye rubbing exerts a mechanical influence on the cornea, which may result in KC [50–52].*  *The differences in reported KC prevalence values may also be due to study design variations. Hospital-based or multicenter studies are typically retrospective, and diagnoses are based on data obtained through routine examinations [8–10]. Studies using registry data [11–13,19] may have inclusion criteria similar to those of hospital-based studies conducted by ophthalmologists. These study designs likely exclude patients with early-stage KC who have not received medical consultation. Therefore, a population-based design may be better for detecting the actual prevalence of KC in the general population. Our study was population-based, and included relatively older subjects aged ≥ 35 years. This method may be effective to detect the actual prevalence because KC may have already developed until 35 years. If the younger subjects were included in the study, the surveyors may miss pre-disease stage patients.*  *Differences in examination instruments must also be considered when determining the prevalence of KC. Before the 1990s, KC was mainly diagnosed using slit lamp findings (such as Fleisher’s ring, Vogt striae, and corneal thinning), keratometry, and retinoscopy. Therefore, it was challenging to detect early-stage KC. The low KC prevalence in studies published during that period may be due to low diagnostic precision and low sensitivity [8–10, 20]. Afterward, Placido-based corneal topography enabled clinicians to detect irregular astigmatism of the anterior corneal surface in detail [25], and corneal tomographers based on slit-scanning or Scheimpflug cameras provided additional information regarding the cornea’s posterior surface and thickness [26–36]. As a result, the prevalence of KC reported in studies using corneal topography or tomography [14,16,17] is higher than that obtained in previous studies due to the higher sensitivity of the instruments.*  *Few Japanese studies regarding the prevalence of KC have been reported. Tanabe et al. reported a very low prevalence of KC (9 per 100,000) [20]. This finding contradicts that reported in this study (796 per 100,000). However, this study was population-based, whereas the study by Tanabe et al. was hospital-based study. The results of hospital-based investigations may be biased toward moderate or severe cases of KC. While the previous studies did not include the diagnostic criteria or apparatus used in the diagnoses, corneal topography or tomography was not performed at the time of the study. In contrast, corneal tomography using AS-OCT, which allows detection of mild changes in tomographic abnormalities in the early stages of KC, was used in this study [30,31].*  *The characteristics of tomographic maps of both eyes in KC or KCS or a control participant were analyzed in this study. In the axial power maps, the inferior steepening pattern was specific for KC and KCS (Table 3). In the anterior and elevation maps, the central regular ridge pattern was dominant in the non-KC (81.0%) and KCS groups (35.0%). Similarly, the central regular ridge pattern was dominant in the non-KC (56.9%) and KCS groups (30.0%) in the posterior elevation maps. In the pachymetric maps, the control participants demonstrated a central round pattern, and the paracentral patterns were dominant in the KC or KCS eyes. To the best of our knowledge, this is the first paper that evaluated corneal tomographic patterns in a population-based study. It must be worth understanding the tomographic patterns of KC/KC for detecting them and also for the differential diagnosis in clinical practice.*  *In KC, sex difference has been discussed. While some studies have reported that the prevalence of KC is higher in male patients [8,10,11,13,14], other studies have shown that the prevalence is not associated with sex [9,16–19]. Jonas et al. reported an association between high-risk KC and the female sex [15]. In our study, no sex differences were found in KC/KCS. In addition, there were no differences in corneal power, cylinder, CCT, or TCT (as evaluated using the Mann–Whitney test; data not shown) between male and female participants with KCS or KC. The sex-specific difference in KC may remain controversial.*  *In the risk investigations of KC/KCS, the values including IOP, CCT, TCT, corneal power, cylinder, asymmetry, HOI, and posterior corneal indices, were associated with KC and KCS in this study, consistent with previous studies [3,16–18].*  *In general, data of one eye of a patient were compared with data of affected and unaffected eyes of other individuals to avoid bias associated with a symmetry in laterality. However, patients with KC have obvious asymmetry in infero-superior differences in a single eye and IEDs. Zadnik et al. previously demonstrated IEDs in visual acuity and corneal cylinder [4], while Chopra reported differences in spherical error and the spherical equivalent [5]. In addition, Henriquez et al. and Dienes et al. found differences in the corneal cylinder, CCT, TCT, and posterior elevation of the cornea using Scheimpflug camera imaging [6, 7]. While these studies were all regarding KC, the data in this study suggest that IEDs in corneal indices are associated with both KC and KCS.*  *The diagnostic instruments used for the diagnosis of KC/KCS vary among studies. A discrimination model optimized for the study-specific purpose and environment is required for minimal and precise screening. In addition, model parameters should be generalizable and easily implemented in clinical settings. While the OCT model evaluated in this study yielded the best precision for detecting KC and KCS (100% sensitivity and 96% specificity), AS-OCT is not widely used in clinics. In contrast, the addition of IED data improved the accuracy of the non-OCT model (from 90% to 95% sensitivity and 79% to 83% specificity), thereby increasing the AUC from 0.876 to 0.940. While eyes with KC in this study population were effectively discriminated in all models, non-OCT + IED is preferable for screening KCS when corneal tomography is unavailable.* |
| Generalisability | 21 | Discuss the generalisability (external validity) of the study results | *28* | *As recent advances in corneal crosslinking have induced a paradigm shift in treating KC, an early diagnosis is increasingly beneficial. In addition, the high incidence of KC in the general population suggests the importance of screening for KC before performing LASIK, placement of phakic IOL, and refractive cataract surgery [36–39,55–57]. Toric, extended depth-of-focus, and multifocal IOL implantation should be avoided in keratoconic eyes to prevent suboptimal results due to corneal HOAs and postoperative refractive errors. If corneal topography or tomography is unavailable in the clinic, IEDs in corneal power, cylinder, and axial length can be used as screening tools for suspected KC* |
| Other information | |  | | |
| Funding | 22 | Give the source of funding and the role of the funders for the present study and, if applicable, for the original study on which the present article is based |  | *None.* |

*Give information separately for cases and controls in case-control studies and, if applicable, for exposed and unexposed groups in cohort and cross-sectional studies.

**Note:** An Explanation and Elaboration article discusses each checklist item and gives methodological background and published examples of transparent reporting. The STROBE checklist is best used in conjunction with this article (freely available on the Web sites of PLoS Medicine at http://www.plosmedicine.org/, Annals of Internal Medicine at http://www.annals.org/, and Epidemiology at http://www.epidem.com/). Information on the STROBE Initiative is available at www.strobe-statement.org.
