# Supplementary material for: Prevalence of keratoconus and keratoconus suspect, and their characteristics on corneal tomography in a population-based study
Source: PLoS One. 2025 Jan 16;20(1):e0308892. doi: 10.1371/journal.pone.0308892 (PMC11737720; doi:10.1371/journal.pone.0308892)
Supplement: Attachment — Submitted filename: Response to Reviewers.docx [file pone.0308892.s002.docx]

**The responses to the comments of the reviewers**

**Journal Requirements:**

*When submitting your revision, we need you to address these additional requirements.*

*1. Please ensure that your manuscript meets PLOS ONE's style requirements, including those for file naming. The PLOS ONE style templates can be found at*

*https://journals.plos.org/plosone/s/file?id=wjVg/PLOSOne_formatting_sample_main_body.pdf and*

*https://journals.plos.org/plosone/s/file?id=ba62/PLOSOne_formatting_sample_title_authors_affiliations.pdf*

**RESPONSE:**

Thank you for the comment, and we revised our manuscript to meet PLOS ONE's style requirements (title page, paragraphs, etc.).

*2. We note that you have included the phrase “data not shown” in your manuscript. Unfortunately, this does not meet our data sharing requirements. PLOS does not permit references to inaccessible data. We require that authors provide all relevant data within the paper, Supporting Information files, or in an acceptable, public repository. Please add a citation to support this phrase or upload the data that corresponds with these findings to a stable repository (such as Figshare or Dryad) and provide and URLs, DOIs, or accession numbers that may be used to access these data. Or, if the data are not a core part of the research being presented in your study, we ask that you remove the phrase that refers to these data.*

**RESPONSE:**

Thank you for your point out. The associations of sex differences with ocular parameters are not the focus of our research. Therefore, we deleted the sentence containing the phrase "data not shown". We revised our paper as below:

*(Lines 388- 392 in Revised Manuscript with Track Changes)*

*“In our study, no sex differences in prevalence were found in KC/KCS. The sex-specific difference in KC may remain controversial.”*

**Comments to the Author**

3. Have the authors made all data underlying the findings in their manuscript fully available?

*Reviewer #1: No*

*Reviewer #2: No*

**RESPONSE:**

My apologies, it seems that the file we uploaded was incomplete. So we revised and uploaded the files again, separating them into eye- and person-specific data. And we revised our paper as below:

*(Lines 193 - 195 in Revised Manuscript with Track Changes)*

*“Our data are available at: https://doi.org/10.6084/m9.figshare.26039557.”*

5. Review Comments to the Author

***Reviewer #1:*** *The authors aimed to determine the prevalence of keratoconus (KC), investigate the corneal tomographic features of KC and keratoconus suspect (KCS) using corneal tomography, and develop predictive models for KC and KCS based on corneal tomography, comparing those with and without corneal tomography. However, there are methodological errors in this study, and it needs improvement.*

*1. The identification of participants in this study is referenced to an article from 1999. However, it was stated that data from 2015-2016 and 2017 were obtained and evaluated after mentioning this. It is essential to provide a clear explanation of the population included in the study.*

**RESPONSE:**

Thank you for this point out, and I apologize for the confusing wording. The basic structure of the study, including the division of the health examination schedule and the method of consent, follows that of the Yamagata study (Funagata study), so the paper from 1999 is also included in the references. The subjects of this study and the data obtained were newly examined and obtained from 2015 to 2017.

We revised our paper as below:

*(Lines 96 - 105 in Revised Manuscript with Track Changes)*

*“This study was conducted as part of the Yamagata Study, a population-based epidemiological investigation examining systemic and ophthalmologic disorders in Japanese individuals aged ≥ 35 years [40–44]. Present research was performed in accordance with the residual medical checkups in Funagata town which was divided into three areas: Funagata, Horiuchi, and Nagasawa areas in terms of schedule. Systemic and ophthalmic data included in this research were collected from residents of Funagata area on June 14 and 28, 2015; Horiuchi area on June 4 and 19, 2016; and Nagasawa area on June 4, 2017.”*

*2. In the literature, there is a population-based keratoconus prevalence study using corneal tomography.**

**RESPONSE:**

Thank you for the comment, and I apologize for the confusing wording again. We are not thinking that this is the first population-based study using tomography, but that this may be the first paper that color map “patterns” of the anterior and posterior surface and corneal thickness from tomography have been evaluated in a population-based design.

We revised our paper as below:

*(Lines 379 - 383 in Revised Manuscript with Track Changes)*

*“The population-based evaluation of the color map patterns of anterior and posterior corneal surfaces may be a unique feature of this study.”*
