# Supplementary material for: Prevalence of keratoconus and keratoconus suspect, and their characteristics on corneal tomography in a population-based study
Source: PLoS One. 2025 Jan 16;20(1):e0308892. doi: 10.1371/journal.pone.0308892 (PMC11737720; doi:10.1371/journal.pone.0308892)
Supplement: Attachment — Submitted filename: 240713_Response to Reviewers.docx [file pone.0308892.s003.docx]

Dear Prof. Timo Eppig,

Thank you for the thoughtful and constructive feedback you provided regarding our manuscript titled “Prevalence of keratoconus and keratoconus suspect, and their characteristics on corneal tomography in a population-based study.”

We revised our paper as you mentioned, I think. We hope that you find the current version of the manuscript suitable for publication.

Sincerely,

Hiroyuki Namba

**The responses to the comments of the reviewers**

I'm sorry for the lack of responses to the comments #3-#7. We were unable to receive the comments due to a technical error.

**Reviewer #1:**

*3. Keratoconus suspect is defined as the presence of keratoconus patterns on the anterior axial power map. After the development of corneal tomography devices, it is accepted that keratoconus develops from the posterior surface of the cornea. In this case, keratoconus suspect should primarily involve changes in the posterior surface patterns. The current definition might have missed keratoconus-suspected patients*

**RESPONSE:**

Thank you for the comment. In our research, we involved changes in both the anterior and posterior surfaces of the cornea as KC and KCS. Cases with only posterior surface changes are defined as forme fruste keratoconus (FFK) and are not included in our study. The reason for this is that there were only a few cases of FFK in this study, and they were either KC or KCS. There were no cases with only posterior changes in both eyes. We revised our paper as below:

*(Lines 137-140 in Revised Manuscript with Track Changes)*

*“KCS was defined as eyes that had visual acuity 20/20 or better and keratoconus patterns in the anterior and posterior corneal surfaces (Fig 4) [3,31-34]. Because there were few eyes with forme fruste keratoconus, which has irregularity only on the posterior surface, we did not include them.”*

*4. The mean age of patients is considerably higher than the typical age of keratoconus diagnosis. Were the patients diagnosed with keratoconus in this study newly diagnosed cases who had not received a diagnosis before the study? The presence of patients who had undergone treatments like CXL is not specified in the study.*

**RESPONSE:**

Thank you for your comment. We included KC whether newly diagnosed or previously diagnosed. One eye that had previously undergone a keratoplasty was excluded as a case with insufficient data in the ocular examination. Eyes that had undergone CXL were not included. CXL is not yet widely used in rural areas of Japan. We revised our paper as below:

*(Lines 4- 9 in Figure 2)*

*“Insufficient data (missing, error, decentering, eyelid interferences, and abnormal corneal shape except for KC), an eye that has undergone a keratoplasty, and 2 eyes with pellucid marginal degeneration (n = 316)”*

*5. It is recommended to remove soft and rigid contact lenses at least 1 week before keratometric measurements. Were the patients' contact lenses removed before measurements?*

**RESPONSE:**

Thank you for your point out. It is correct and we would have done the same if we could have. However, due to the limitation of a joint medical checkup project with the Department of Internal Medicine, and because prior restrictions on contact lens use would lead to inconvenience in daily life, we requested it, but it was difficult to enforce it.

*6.As a result of the study, using axial length in keratoconus suspect cases is not a clinically appropriate approach.*

**RESPONSE:**

Thank you for your comment. You raise a valid point, and the axial length measurement may not be accurate in KC with corneal shape abnormalities. However, in the investigations of KC risk assessment, our goal is to use the data obtained from clinical examinations to make a diagnosis of KC. I think the accuracy of axial length measurement in KC may not be an essential issue.

*7. Determining sensitivity and specificity values in a study with 822 participants, including 7 keratoconus and 12 keratoconus-suspected patients, will not provide objective results.*

**RESPONSE:**

I think you have a valid point. I think the number of cases may not be sufficient to calculate an epidemiologically accurate prevalence. However, the prevalence of KC in Japan has not been adequately studied in the past, and it is relatively close to the prevalence in China also in East Asia, therefore, I think we were able to show some significance. We indicated the limitation on the line 432-433.

**OPTIONAL:**

We unified the terms "person-wise" and "eye-wise" into "person-specific" and "eye-specific" as below:

*(Lines 206 in Revised Manuscript with Track Changes)*

*“Table 1. Person-specific characteristics in non-KC, KCS, and KC subjects.”*

*(Lines 222 in Revised Manuscript with Track Changes)*

*“Table 2. Eye-specific characteristics in non-KC, KCS, and KC subjects.”*
